# Characteristics, Risk Factors, and Survival Analysis of *Candida auris* Cases: Results of One-Year National Surveillance Data from Oman

**DOI:** 10.3390/jof7010031

**Published:** 2021-01-07

**Authors:** Azza Al-Rashdi, Amal Al-Maani, Adil Al-Wahaibi, Abdullah Alqayoudhi, Amina Al-Jardani, Seif Al-Abri

**Affiliations:** 1 Central Public Health Laboratories, Directorate General for Disease Surveillance and Control (DGDSC), Ministry of Health, Muscat 393, Oman; aksaljardani@gmail.com; 2 Directorate General for Disease Surveillance and Control (DGDSC), Ministry of Health, Muscat 393, Oman; adilwahaibi@gmail.com (A.A.-W.); alqaudi99@gmail.com (A.A.); salabri@gmail.com (S.A.-A.)

**Keywords:** candida, *C. auris*, survival, risk factors, fatality rate, resistance

## Abstract

Background: *Candida auris* (*C. auris*) is an emerging healthcare-associated pathogen resulting in significant morbidity and mortality. The aim of this study is to report data from the national *C. auris* surveillance system for 2019 and conduct a survival analysis of the reported cohort. Methods: a retrospective analysis was conducted for all *C. auris* cases reported nationally to the Oman Antimicrobial Surveillance System (OMASS) in 2019, and isolates were sent to the Central Public Health Laboratories (CPHL). Clinical and demographic data were obtained through the E-Surveillance reporting system and the Electronic System (NEHR Al-Shifa) at CPHL. Statistical analysis was done using Kaplan–Meier analysis and Cox proportional hazard models. Results: One hundred and twenty-nine isolates of *C. auris* were grown from 108 inpatients; 87% were isolated from clinical samples, of which blood was the most common (38.9%). Forty (37%) were ≥65 years of age, 72 (66.7%) were males, and 85 (78.7%) were Omani nationals. Of the total isolates, 43.5% were considered as colonization; 56.5% were considered infection, of which 61.8% of them were candidemia. At least one risk factor was present in 98.1% of patients. The mean time from admission to infection was 1.7 months (SD = 2.8), and the mean length of hospital stay was 3.5 months (SD = 4). Totals of 94.8% and 96.1% of the isolates were non-susceptible to fluconazole and amphotericin, respectively. The variables found to be significantly associated with longer survival post *C. auris* diagnosis (*p* < 0.05) were age < 65 years, absence of comorbidities, length of stay < 3 months, colonization, and absence of candidemia. The infection fatality rate was 52.5%. Conclusion: Including *C. auris* in an ongoing antimicrobial surveillance program provides important data for the comprehensive management of this growing public health threat. The current study shows health care outbreaks of *C. auris* are ongoing, with 52.5% infection fatality, although our isolates remained sensitive to Echinocandins in vitro.

## 1. Introduction

*Candida auris* (*C. auris*) emerged as a resistant fungal pathogen for the first time in 2009. Since then, it has spread to all continents but Antarctica [1,2]. It is increasingly described as an agent of hospital-acquired infections and has gained global attention due to its multidrug-resistant properties and high crude mortality rate, up to 40% in cases of bloodstream infections [2,3,4,5]. In addition, *C. auris* strains are difficult to identify and can be misidentified as other phylogenetically related pathogens [6]. In Oman, the first report of *C. auris* isolates was in 2017, in which five cases were described with bloodstream infection [7]. After introducing diagnostic facilities, further hospital clusters were identified, and their control was a major challenge to the existing infection prevention and control measures [8]. In 2017, the Oman Antimicrobial Resistance Surveillance System (OMASS) included *C. auris* as an emerging pathogen at the national level, and a surveillance system was put in place to monitor the clinical, epidemiological, and drug susceptibility profiles to further guide the antimicrobial stewardship program and other AMR-related activities and interventions [9,10]. The Global Antimicrobial Resistance Surveillance System (GLASS) also includes invasive fungal bloodstream infections (BSIs) caused by the *Candida* species as part of the priority pathogens monitored for the AMR global action plan [11].

The threat of *C. auris* evolution to pan-resistance has become a reality and has been reported from multiple countries, resulting most of the time in fatalities [3,4,5,12,13]. This emphasizes the importance of monitoring this pathogen and its impact on morbidity and mortality. This study reports OMASS *C. auris* surveillance data from 2019 and conducts a survival analysis for infected/colonized patients reported during this year.

## 2. Materials and Methods

All cases of *C. auris* that had been entered into the national electronic surveillance system (E-Surveillance) and *C. auris* isolates received at the Central Public Health Laboratories (CPHL; the reference laboratory for the Antimicrobial Resistance Surveillance System) for confirmation and antimicrobial susceptibility testing (AST) between January and December 2019 were included in this study. These included all isolates from clinical and screening samples. Patient screening was conducted according to the national policy of infection prevention and control for *C. auris*. In general, screening is recommended for patients coming from affected hospitals locally, nationally, or internationally; patients admitted to units with newly identified and/or ongoing cases or colonization; and close contacts of newly diagnosed patients in intensive care or high-dependency units. All patient duplicates for the same episode of admission were removed, even if more than one positive sample was involved, considering only the most invasive sample. These duplicates were included when the interval between episodes of infection/colonization was ≥2 weeks for the same patient, which follows the national surveillance protocol for Multidrug-Resistant Organisms.

### 2.1. Clinical & Demographic Data

Clinical and demographic data were collected using the E-Surveillance reporting system and the Electronic System (NEHR Al-Shifa) at CPHL. The data included patient age, gender, nationality, diagnosis, ICU admission, risk factors, length of hospital stay, antifungal agents received and outcome, and specimen type. Risk factors included comorbidities such as hypertension (HTN), diabetes mellitus (DM), cardiovascular disease (CVD), chronic kidney disease (CKD), and immunodeficiency as well as presence of artificial ventilation, tracheostomy, indwelling catheter/device, ICU stay, and administration of multiple antibiotic courses during hospital stay. Indwelling catheters/devices included central venous line, external ventricular device (EVD), ventriculo-peritoneal (VP) shunt, percutaneous endoscopic gastrostomy (PEG) tube, and intracutaneous chest tube (ICD). The infection origin (hospital-acquired vs. community-acquired) was determined using the difference between the first positive specimen collection date (infection date) and admission date, which was further verified by the infection control team in the facility. Hospital-acquired infection was defined as infection occurring ≥ 48 h after admission or after transfer from another healthcare institution, otherwise it was labeled as community-acquired infection.

The infection fatality rate was calculated as the number of infected patients who died out of the total number of infected patients.

### 2.2. Identification & AST

Isolates were identified using MALDI-TOF (Maldi Biotyper MBT Compass 4.1.100, Bruker Daltoniks GmbH, Bremen, Germany) or Vitek 2 version 8.01 using a YST ID card. AST was done using Vitek 2 with the AST-YS07 card, which includes fluconazole, voriconazole, caspofungin, micafungin, amphotericin, and flucytosine. Susceptibility was interpreted according to CDC tentative breakpoints for Fluconazole, amphotericin, and echinocandins [14].

### 2.3. Data Analysis

The basic characteristics of the patients were analyzed using percentages (for categorical variables) and medians with interquartile ranges (for continuous variables). Age and length of stay were categorized into two factors each: young < 65 and old ≥ 65 for age; <3 months or ≥3 months for length of stay.

Survival times were estimated by Kaplan–Meier analysis, with the start point as the specimen collection date and the endpoint as the date of death. Univariate factors (age group, nationality, hospital length of stay, hospital, presence of comorbidities, presence of candidemia, and colonization versus infection) were compared using the log rank test. The simultaneous influences of these factors on survival time were examined using Cox proportional hazard models. All analyses were performed using R software version 4.0.

The study was approved by the Directorate General for Disease Surveillance and Control. Informed patient consent was not required because we utilized anonymous data collected for public health purposes, and no personal information was shared.

## 3. Results

A total of 129 *C. auris* isolates were identified in 108 patients during the study period. Eighteen patients had more than one episode of *C. auris*, and no duplicates needed to be removed according to the inclusion criteria. All patients were admitted to a hospital. Out of these isolates, 16 (12.4%) were screening samples obtained for infection control purposes, and 113 (87.6%) were clinical samples including 44 (38.9%) blood, 41 (36.3%) urine, 9 (8%) respiratory, 9 (8%) central line tip, 7 (6.2%) wound, and 3 (2.6%) other samples.

Among the 108 included patients, 40 (37%) were ≥65 years of age, 72 (66.7%) were males, and 85 (78.7%) were Omanis. The isolate was classified as colonization in 47 (43.5%) and infection in 61 (56.5%) patients, with 42 (68.8%) of them having candidemia. The mean time from admission to infection was 1.7 months (SD = 2.8) (Table 1). At least one risk factor was present in 106 of the infected/colonized patients: 74 had other comorbid diseases, 42 had an indwelling catheter/device, 41 had tracheostomy, 85 were on ventilators, 91 received multiple courses of antibiotics during their hospital stay, and 85 patients were admitted to the ICU at some point during hospital stay. The mean length of hospital stay for the included patients was 3.5 months (SD = 4).

In addition, of the 18 patients with multiple episodes of *C. auris*, 3 patients acquired infection followed by persistent colonization. On the other hand, 4 patients were found positive for *C. auris* in the screening samples and developed infection several days to several weeks after the initial screening.

Out of 53 infected patients with available treatment data, 47 received antifungal treatment (63.8% Echinocandins, 23.4% Azoles, 4.3% Amphotericin, and 8.5% multiple antifungal agents), and 15/49 died while on therapy. There were 6/53 patients who were not started on antifungal (4 died prior to results, 2 improved with source control). The overall fatality rate for the infected patients was 52.5%.

AST was conducted for 77/129 isolates (59.7%), and results are shown in Table 2. Echinofungins (caspofungin and micafungin) showed the lowest MIC among the antifungal classes tested, with 98.7% and 97.4% of tested isolates having MICs of <1 µg/mL and ≤1 µg/mL for caspofungin and micafungin, respectively. Fluconazole and amphotericin were found to have high MICs for the majority of tested isolates: 94.8% and 96.1%, respectively.

The infection origin was classified as healthcare for all patients in this report; all patients were infected/colonized ≥ 48 h after admission (100), or, in eight patients, *C. auris* was detected during routine screening, when transferred from another facility or readmitted after recent discharge from the same facility, taking into account the first *C. auris* isolate for each patient.

**Figure 1:**
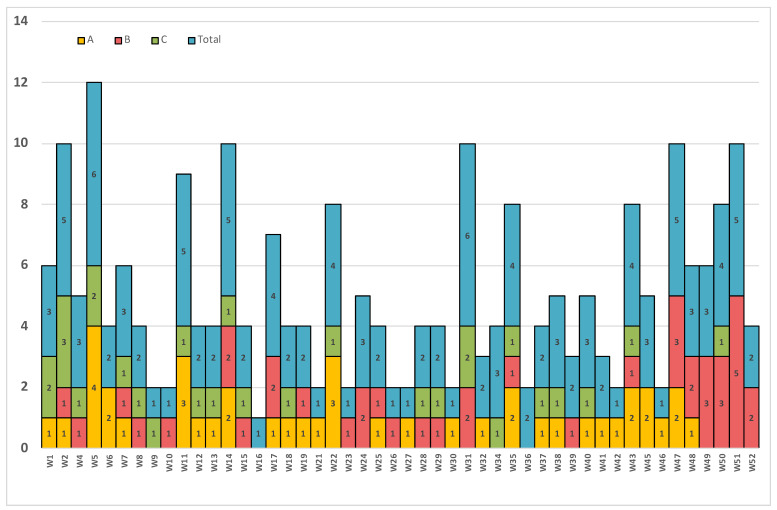
*C. auris* isolates by week over a 1 year period from (A) hospital A, (B) hospital B, (C) hospital C, and (Total) in all 7 hospitals. Numbers in the columns represent number of C.auris isolates per that week.

The isolates were received from seven hospitals in the country. The majority of these isolates (89.2%) were grown from patients admitted to three major hospitals—A, B, and C (a tertiary medical and surgical center, a tertiary trauma center, and a secondary medical and surgical center)—while the remaining 10.8% were from patients admitted to four other district hospitals. The epidemiological curve in Figure 1 shows the distribution of these isolates throughout the year. It is clear that these clusters were scattered across all months, without clear interruptions for the 3 major hospitals, A, B, and C which might indicate ongoing outbreaks.

**Figure 2:**
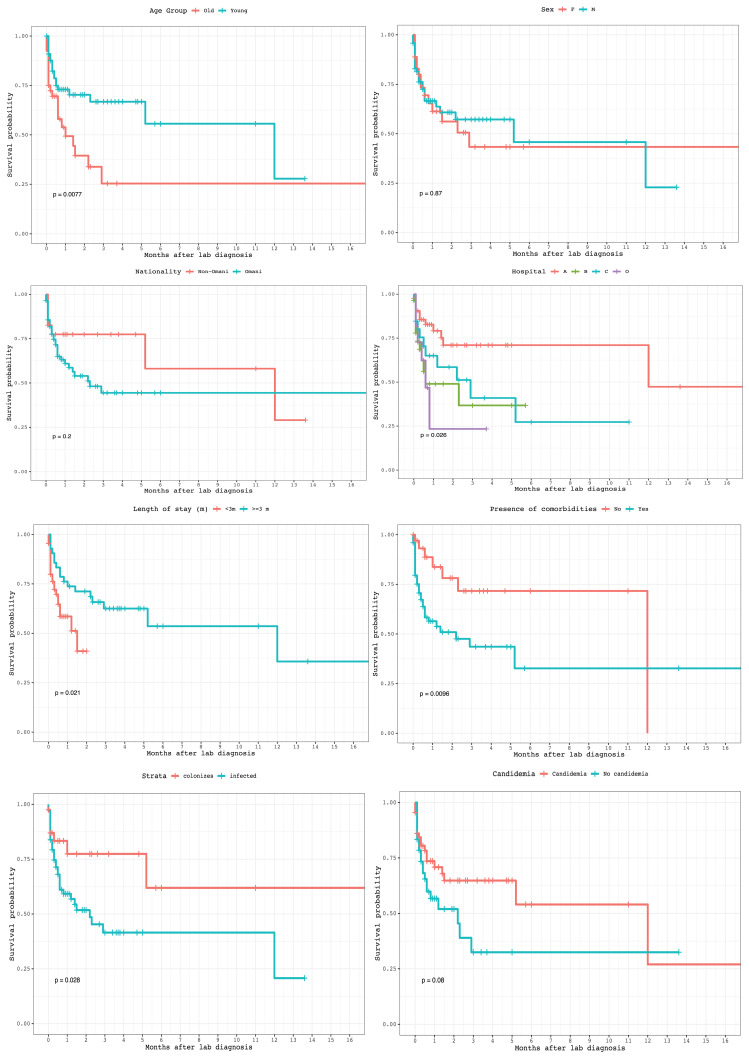
Kaplan–Meier survival analyses of age, sex, nationality, length of stay, presence of comorbidities, hospital where patients were admitted, candidemia, and colonization vs. infection.

Using Kaplan–Meier analysis, we found age < 65 years, absence of comorbidities, hospital A, length of stay < 3 months, colonization, and absence of candidemia were significantly associated with longer survival post *C. auris* diagnosis (*p* < 0.05), whereas the other factors were not (Figure 2).

**Figure 3:**
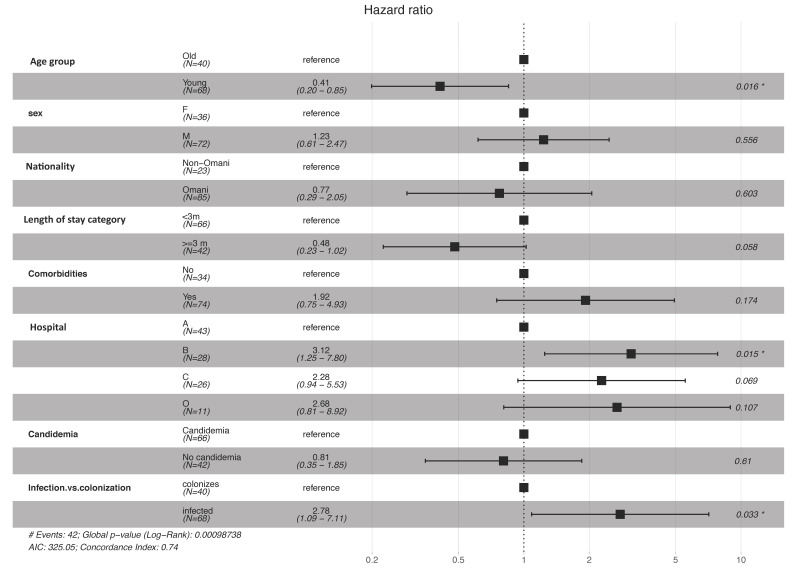
Multivariate Cox proportional hazard model for survival post diagnosis by age, sex, nationality, length of stay, presence of comorbidities, hospital where patients were admitted, candidemia, and colonization vs. infection. Column 3 represent the hazard ratio (95% confidence interval), * statistically significant, *p* value < 0.05.

Adjusting for all studied factors, the multivariate Cox proportional hazard model shows that longer survival post diagnosis was significantly associated with younger age (<65 year) with an adjusted hazard ratio (AHR) of 0.41 (95% CI 0.20–0.85; *p* = 0.016). On the other hand, worse survival post diagnosis was found in infection compared to colonization groups (AHR 2.78, 95% CI 1.09–7.11, *p* = 0.033) and hospital B compared to A (AHR 3.12, 95% CI 1.25–7.80, *p* = 0.015; Figure 3).

## 4. Discussion

The current study describes the characteristics and outcomes of patients infected/colonized with *C. auris* at the national level from the OMASS data of 2019. Data clearly show the high burden and impact of this pathogen as a cause of healthcare-associated infection and ongoing outbreaks in the hospitals. The study underscores the importance of ongoing surveillance as a tool to assess the magnitude, burden, and impact of intervention on this growing healthcare challenge. Although our isolates for *C. auris* were susceptible to Echinocandins in vitro, and no pan-resistance had occurred, the fatality rate remained high at 52.5%.

Although the emergence of *C. auris* was first reported in 2009, a subsequent report identified *C. auris* in a sample collected back in 1996 [15]. Globally, it was under-reported, and it managed to surface as a significant AMR pathogen only in the last 5 years after several reports of health care outbreaks [16,17]. One theory that tried to explain this re-emergence was global warming, which might have led to narrowing the human ‘thermal restriction zone’ that protects humans from infection [18]. Many of the reported delays in the identification and mismanagement of patients are related to diagnostic challenges in identifying isolates and differentiating them from other *Candida* species [19,20]. At the national level, *Candida* isolates are diagnosed and subtyped at CPHL and some tertiary hospitals in the capital city of Muscat, but the delay in transporting samples from hospitals in other districts may have resulted in delaying appropriate intervention and increased the burden and fatalities.

All candida isolates from sterile sites should be identified at the species level to guide antifungal therapy [21]. In addition, the same should be done for candida isolated from non-sterile sites for infection control and surveillance purposes, as recommended by the Centers for Disease Control and Prevention (CDC) [22]. To differentiate *C. auris* from other candida species, culturing at 42 C or higher has been used [19]. Nevertheless, more accurate methods of identification are always required. Among these, MALDI-TOF and Vitek 2 YST ID card are accurate methods when *C. auris* is added to their databases [21,23]. In addition, PCR, real-time PCR [24,25,26], and sequencing methods have been evaluated and give excellent results to differentiate *C. auris* from other candida species. All *C. auris* isolates in our study were identified using either MALDI-TOF or Vitek 2 YST ID card, which are available in our reference laboratory. Early identification and reporting of *C. auris*, due to the availability of diagnostic tests, will improve AMR surveillance and better patient outcomes in many low- and middle-income countries. It will also provide better insight to the magnitude and burden of infection, which will impact global intervention plans [27].

A number of studies determined different possible risk factors for developing *C. auris* infection/colonization. These included the presence of tracheostomies, PEG tubes, and ventilators; prior antibiotic use; ICU stay; and comorbidities such as DM, CKD, and lung disease [28,29,30,31,32,33]. In our study, the majority of these patients had a tracheostomy (38.0%), indwelling catheters (38.9%), comorbidities (68.5%), were on a ventilator (78.7%), admitted to the ICU (78.7%), and/or received multiple courses of antibiotics during their hospital stay (84.3%). These factors are significant to consider when candida infection is suspected in order to guide management and infection control measures.

Although the hospitals in our study that reported *C. auris* are acute care institutions, they also provide long-term nursing care for cases involving stroke and multiple traumas because there is an absence of such long-term healthcare facilities in the country. This was reflected by the prolonged mean length of hospital stay of 3.5 months, which in turn affected the survival of these patients.

The reported mortality rates attributable to invasive *C. auris* infection range from 30% to 59% globally [31,34]. Crude in-hospital mortality rates for *C. auris* candidemia are estimated to range from 30% to 72% [20]. The overall infection fatality rate in our study was 52.5%, which was similar to the figure mentioned earlier from another study in Oman (53.1%) [8]. The majority of these patients required ICU admission.

The survival analysis showed that statistically significant factors negatively attributed to survival post diagnosis were old age, presence of comorbidities, *C. auris* infection rather than colonization, long hospital stay, and presence of candidemia. The effect of the latter on survival was analyzed and found to be statistically significant by Sayeed et al., while there was no difference in mortality between infected and colonized patients [33].

Our data clearly show there were ongoing outbreaks in three out of the seven hospitals reporting *C. auris* cases. The outbreak control of *C. auris* requires implementing multiple infection control measures, as mentioned in a previous report by Taori et al. [35] Despite the measures implemented in these three hospitals, the outbreaks were ongoing throughout the year, which clearly shows the difficulty in eradicating *C. auris* and the burden this organism can put on infection control programs at any healthcare facility.

Regarding antimicrobial susceptibility testing, there are no specific Breakpoints identified for *C. auris*; tentative breakpoints developed by CDC are currently in use [14]. Apart from the study by Kellie Arensman et al. showing a low rate of resistance to fluconazole (14%) and amphotericin B (3.6) [36], many reports detected a high rate of resistance to these antifungal classes. A recent review of *C. auris* cases from the Middle East showed in vitro antifungal susceptibility testing identified 100% of the strains to be resistant to fluconazole (MIC 32 ≥ 256 mg/L) while having variable resistance to other antifungal agents [37]. This is similar to reports by Ruiz-Gaitán et al. and Sayeed et al. [32,33]. A report from the USA showed that >90% of *C. auris* isolates were resistant to fluconazole, >60% were resistant to amphotericin, and 3.9% were resistant to echinocandins; three isolates were found to be pan-resistant (resistant to the three antifungal classes) [13]. Lockhart et al. found that 4% of the isolates studied were pan-resistant, involving patients from Pakistan, India, South Africa, and Venezuela [34].

According to our results, the isolates of *C. auris* were not susceptible to fluconazole, and only 5.2% had an MIC of ≤8 µg/mL. Similar findings were observed with amphotericin: only 3.9% of the isolates had an MIC of <1 µg/mL. On the other hand, the majority of isolates were susceptible to echinocandins (caspofungin and micafungin), with 98.7% and 97.4% having MICs of <1 µg/mL and ≤1 µg/mL, respectively. It is reassuring that our isolates remained sensitive to the Echinocandins group. This was similar to the above reports, but it differs from a report in India where 40% of the isolates were not susceptible to caspofungin [38].

The fact that 15 patients died while on antifungal treatment raises the possibility of treatment failure despite the MIC results in vitro. In analyzing 11 of these cases, for which AST data were available, 4 received antifungal agents with MICs in the non-susceptible range. A striking finding was that the remaining seven patients received echinocandins, and their *C. auris* AST results showed MICs to Echinocandins in the susceptible range. Acquiring resistance to echinocandins while on echinocandins has been reported previously [22]. However, this could not be proven here because no repeated samples taken with *C. auris* isolated and AST done for these patients while on treatment. It is still unclear how well in vitro AST results correlate with the in vivo performance of antifungal agents against *C. auris* infection. However, we must take into account other possible causes of death, such as underlying comorbidities and uncontrolled source of infection.

A limitation of this study is that it is retrospective and is based on voluntary reporting by the hospitals. In addition, no genotyping was performed to analyze the virulence and genetic background of its antifungal resistance. However, a previous report from Oman identified *C. auris* isolates as part of clade 1 (South Asian), and Muñoz et al. reported different resistance genes in this clade and other clades [8,39]. In addition, the small sample size might have affected the results and led to wide CIs, which might possibly explain the absence of significant *p*-values in some variables.

## 5. Conclusions

In conclusion, *C. auris* is an additional burden to the healthcare system and is the leading antimicrobial-resistant fungi. *C. auris* should be at the top of the AMR agenda because: it prefers the healthcare environment where many high-risk patients with complex health issues are managed; it can persist on medical devices and in the environment; it has developed resistance to many antifungals; pan-resistance is possible, and we are running out of treatment options; and it has high crude mortality rates. A comprehensive intervention program is urgently needed to decrease the burden of *C. auris*, and it should include an ongoing surveillance program that covers clinical, epidemiological, and drug susceptibility patterns on a regular basis, thereby strengthening the infection prevention and control programs in acute care hospitals. In addition, the fact that all cases in this cohort were hospital-acquired stresses the importance of antimicrobial stewardship programs in healthcare facilities.

Furthermore, this study emphasizes the importance of establishing a molecular genotyping service, such as Whole-Genome Sequencing, at CPHL for *C. auris* as part of the diagnostic toolkit during outbreak investigations and for surveillance. In addition, these data show the need to establish diagnostic services for *C. auris* at the district level.

## Figures and Tables

**Table 1:** Descriptive statistics of *C. auris* cases in 2019.

Categories	Overall (N = 108)
Age group: ≥65 years	40 (37%)
Males	72 (66.7%)
Omani	85 (78.7%)
Candidemia	42 (38.9%)
Infection (not colonized)	61 (56.5%)
Length of stay (months): <3 m	66 (61.1%)
Comorbidities	74 (68.5%)
Length of stay (months) after diagnosis: Mean (SD)	1.7 (2.8)
ICU admission	85 (78.7%)
Died (out of total patients)	42 (38.9%)

**Table 2:** *C. auris* isolate (N = 77) distributions among different MIC values for the antifungal agents tested.

Antifungal	Fluconazole	Voriconazole	Caspofungin	Micafungin	Amphotericin B	Flucytosine
MIC	≤8 µg/mL 4 (5.2%)	≤1 µg/mL 68 (88.3%)	<1 µg/mL 76 (98.7%)	≤1 µg/mL 75 (97.4%)	<1 µg/mL 3 (3.9%)	≤8 µg/mL 70 (90.9%)
MIC	16 µg/mL 34 (44.2%)	2 µg/mL 7 (9.1%)	1 µg/mL 0 (0.0%)	2 µg/mL 0 (0.0%)	1 µg/mL 9 (11.7%)	16 µg/mL 1 (1.3%)
MIC	≥32 µg/mL 39 (50.6%)	≥4 µg/mL 2 (2.6%)	≥2 µg/mL 1 (1.3%)	≥4 µg/mL 2 (2.6%)	≥2 µg/mL 65 (84.4%)	≥32 µg/mL 6 (7.8%)

## Data Availability

Data in this study is available on request.
